# Mr. Davies's Case of Spontaneous Ptyalism

**Published:** 1811-12

**Authors:** H. Davies

**Affiliations:** Piccadilly


*To the Editors of the Medical and Physical Journal.*

GENTLEMEN,

IF the following "Case of excessive Ptyalism without the exhibition of Mercury" be deemed worthy of insertion, you will have the goodness to assign it a page in your useful Journal.

Mrs. C. about 45 years of age, who for a few years past has been confined to her bed in consequence of loss of nervous influence, disabling her from standing or walking, was, during the course of my attendance on her, some time ago, labouring under a most distressing ptyalism, for ten days or a fortnight, insomuch that she spat daily two or three pints of viscid fluid. Not knowing exactly the source of so unusual a symptom, without any excitement from mercury, I could only trace it to the general debility of the system; therefore prescribed tonic remedies, with occasional aperients. Little or no relief being obtained by this plan; and, anxious to know if any other might prove more efficacious, recourse was had to the opinion of a physician of eminence. On my previously mentioning the history of her case to him, he could not be persuaded but the patient had been tampering with some empirical medicine containing mercury; but, on my applying the question closely on the subject to her, she *positively* avowed to the contrary, with no reason on my part to question her veracity. The physician proceeded on a similar plan of the tonic kind, which I had adopted before; but with no better success, the spitting still continuing as before. Impatient of relief, another physician was consulted, who prescribed a chalybeate plan, fully assuring her she would soon be cured; but this was easier promised than performed. All appeared to be equally inefficacious, the cure remaining in statu quo. The patient at last, disappointed in her expectations, desisted from pursuing the plan any longer, and left off her medicines altogether. However, in a moderate time after, the complaint gradually subsided, and she has had no relapse of it since.

It may be worthy of remark, that the principal relief she obtained during the urgency of the case, was from the aperients before alluded to; for such was the inactive state of the bowels during that period, that she took a mixture containing ℥vj. of sulphate of magnesia daily, to obviate that tendency.

My reason for submitting this cure for insertion in your useful miscellany, is to solicit the observation of some of your ingenious ingenious and intelligent correspondents, respecting the connexion subsisting between the nervous system, in its morbidly deranged state, and the salivary glands of the mouth and throat; for I have since observed, the symptoms alluded to, in a slighter degree, occurring in patients of the same cast.

I am, &c.

H. DAVIES.

*Piccadilly, Oct.* 3, 1811.

